# Does Chaotic Family Relate to Early Externalizing Problems? The Roles of Child Callousness and Mindful Motherhood

**DOI:** 10.3390/bs16101817

**Published:** 2026-10-04

**Authors:** Jiangyang Wang, Ruifeng Tan, Suiqing Chen, Xinkui Chen

**Affiliations:** 1 School of Education, Guangzhou University, Guangzhou 510006, China; 2112308010@e.gzhu.edu.cn (J.W.); tanrf@m.scnu.edu.cn (R.T.); 2 School of Education, South China Normal University, Guangzhou 510631, China; 3 Department of Applied Psychology, Guangdong University of Foreign Studies, Guangzhou 510420, China

**Keywords:** household chaos, callous–unemotional traits, externalizing problem behaviors, maternal mindful parenting

## Abstract

Preschoolers’ externalizing problem behaviors have a profound negative impact on their social adjustment, both concurrently and in the future. Household chaos is a key environmental risk factor; however, the mechanisms underlying this link require further investigation. The current study aimed to examine associations between household chaos and externalizing problem behaviors in preschool children and the roles of callous–unemotional traits and maternal mindful parenting. The participants were 615 children aged 3–7 years (341 boys and 274 girls) and their mothers from Guangdong, China. Data on household chaos, maternal mindful parenting, children’s callous–unemotional traits, and externalizing problem behaviors were collected using mother-reported questionnaires. Results indicated that household chaos was significantly and positively related to externalizing problem behaviors in young children, with callous–unemotional traits acting as a mediator. Maternal mindful parenting was also found to moderate the association between callous–unemotional traits and externalizing problem behaviors. These findings emphasize the necessity of optimizing the home environment and providing mindful parenting training in preventing and intervening in early childhood socioemotional and behavioral problems.

## 1. Introduction

Generally, early human development is inseparable from the influence of the family environment. In line with the bioecological and family systems models, early developmental outcomes are significantly influenced by the familial interaction and household experiences that each child encounters (Bronfenbrenner, 1986; Cox & Paley, 1997). Multiple household experiences (e.g., parental emotional functioning, parenting styles, intimate relationship and household disorganization) can significantly shape children’s cognitive, behavioral, and socioemotional functioning in the present and future (Mills-Koonce et al., 2022). Negative developmental outcomes in early childhood are robustly linked to adverse family dynamics, such as household chaos. In particular, during and after the pandemic, vulnerability and disorganization in family well-being could be highly evident and hinder children’s social adjustment (Prime et al., 2020).

It is important to note that preschool children’s conduct problems have received significant and growing research attention as one of the resulting developmental outcomes of chaotic home environments (Coldwell et al., 2006; Deater-Deckard et al., 2009; Dumas et al., 2005; Larsen & Jordan, 2020; Wilhoit et al., 2021). Preschool is a high-risk period for externalizing problem behaviors (EPBs), including hyperactivity, aggression, defiance and disruptive conduct (Bao et al., 2016). These behaviors often demonstrate developmental continuity (Thompson et al., 2011), with far-reaching consequences that extend into adolescence, compromising academic performance, interpersonal relationships, and overall social adaptation (Moilanen et al., 2010; Olivier et al., 2020). Thus, exploring the potential mechanisms through which chaos in the home contributes to early externalizing problem behaviors has significant practical implications.

The existing literature has largely overlooked individual psychopathological features, such as callous–unemotional (CU) traits, as potential developmental mechanisms, despite their well-established role as risk factors for externalizing problems (Hyde et al., 2013). Similarly, it has been thought that early psychopathology is also associated with family contextual factors. For instance, several studies have demonstrated significant links between household chaos and early childhood psychopathology symptoms (Fisher & Brown, 2018; Fontaine et al., 2011; Mills-Koonce et al., 2016). It is noteworthy that the relationship between chaotic growing environments and children’s psychopathology is not necessarily immutable. Enhanced parental awareness and positive parenting practices among caregivers, especially mothers, may reduce children’s psychopathology symptoms, even within a disordered or chaotic home environment. Accordingly, the mechanisms underlying the link between chaotic home environments and early externalizing problem behaviors require further elucidation.

### 1.1. Household Chaos and Early Externalizing Problem Behaviors

Household chaos is a multifaceted environmental construct characterized by temporal and structural disorganization, crowding, absent routines, disorder, and unpredictability, and it is increasingly conceptualized as a chronic environmental stressor rather than an acute stressor (Bronfenbrenner & Evans, 2000; Corapci & Wachs, 2002; Evans & Wachs, 2010). In line with the Family Stress Model, chronic family stressors such as household chaos are thought to deplete parents’ psychological resources and disrupt parenting practices, which in turn are associated with children’s socioemotional and behavioral adjustment (Conger & Donnellan, 2007). For instance, chaotic home environments have been linked to negative parenting (e.g., hostile parenting), which may lead to later difficulties in children’s social adaptation (Coldwell et al., 2006; Tucker et al., 2018).

In particular, a growing body of evidence indicates a significant positive association between household chaos and early externalizing problems (Deater-Deckard et al., 2009; Dumas et al., 2005; Farbiash et al., 2014; Larsen & Jordan, 2020; Volckaert & Noël, 2018; Wilhoit et al., 2021). Dumas et al. (2005) conducted a study among preschool children and found that household chaos was positively correlated with maternal reports of children’s anger and aggression. Similarly, a longitudinal study by Deater-Deckard and colleagues (2009) showed that chaotic household exposure in kindergarten or first grade predicted a rise in behavioral problems two years later. Indeed, certain structural dimensions of household chaos also demonstrate a clear link to externalizing behaviors. For instance, higher levels of background television noise are related to elevated aggressive behaviors and attention difficulties in early childhood (Martin et al., 2012). Children from families with consistent daily routines and clear organization typically show lower levels of externalizing problems (Bater & Jordan, 2017; Rijlaarsdam et al., 2016).

In addition, children exposed to severe household chaos from an early age may encounter psychological crises and personality trait changes persisting into adulthood (Shiner et al., 2017). For example, a study focusing on adolescents has revealed that exposure to chaotic home life is more likely to lead to delinquent behaviors (Joo & Lee, 2020). However, due to children’s immature minds, exposure to household chaos in early childhood could exert a more profound negative impact. Families of preschoolers experiencing household chaos may face crises and deserve greater attention and support.

Based on the above research, this study hypothesizes that household chaos is positively associated with EPBs in early childhood (Hypothesis 1).

### 1.2. Callous–Unemotional Traits as the Mediator

Developmental psychopathology offers a critical perspective for examining the formative mechanisms of children’s externalizing behaviors. In addition to problems in emotional and behavioral self-regulation, there is another significant pathway involving impaired conscience development marked by an interpersonal orientation devoid of empathy and emotional responsiveness (Frick et al., 2014a). Callous–unemotional traits, conceptualized as low levels of empathy and guilt and reduced concern for others, significantly predict externalizing problems across childhood (Frick & Morris, 2004; Waller et al., 2016). A meta-analysis has shown that CU traits act as a marker of conduct problems in early childhood (Longman et al., 2016). Therefore, CU traits are associated with externalizing behaviors in young children.

Similarly, the second pathway proposed by developmental psychopathology suggests that the expression of CU traits is closely linked to contextual factors. Importantly, CU traits in childhood are considered relatively stable rather than immutable inherent dispositions; their expression is malleable and contextually sensitive (Frick et al., 2014b; Waller et al., 2013). Specifically, the Adaptive Calibration Model (ACM) posits that extreme emotional response patterns, such as CU traits, may function as adaptive strategies for coping with stressful environments (Del Giudice et al., 2012). In an overstimulating and chaotic household, children may exhibit such emotional styles, which are concurrently associated with elevated CU traits (Evans & Wachs, 2010). A study involving preschool and school-age children indicated that children’s CU traits were positively associated with household chaos, regardless of children’s age (Payot et al., 2024). In addition, Fontaine et al. (2011) showed that elevated household chaos at age 4 predicted memberships in the stable-high CU trajectory from 7 to 12 years old, indicating the malleability of CU traits across childhood.

According to the General Aggression Model (GAM), situational factors, such as environmental stress, may influence externalizing behaviors through individual traits (Anderson & Bushman, 2002). It is thought that in chaotic family surroundings, developmental changes in CU traits are more likely to be precursors to externalizing problem symptoms in young children. For example, several previous studies have demonstrated that CU traits serve as a mediator linking negative parenting to early externalizing behaviors (Zhang & Zhu, 2023; Y. Zhao et al., 2024). Furthermore, a prior study has shown that levels of family chaos are concurrently associated with both CU traits and externalizing behavior problems in young children (Mills-Koonce et al., 2016).

Based on the above research, this study proposes that CU traits mediate the relationship between household chaos and EPBs in preschool children (Hypothesis 2).

### 1.3. Mindful Motherhood as the Moderator

Mindful parenting, characterized by interpersonal mindfulness in parenting, refers to intentionally, non-judgmentally, and in the present moment attending to one’s child and engaging in parenting practices (Kabat-Zinn & Kabat-Zinn, 1997). According to the mindful parenting model, such mindful capacity is a psychological resource that helps parents sustain their well-being under stress, which in turn reduces negative child outcomes through responsive parenting strategies and a warm parent–child bond (Duncan et al., 2009). Empirical research has consistently linked mindful parenting to positive child psychosocial development, manifested as increased internal strengths (He et al., 2025; Potharst et al., 2021) and decreased problematic behaviors (Donovan et al., 2022; Park et al., 2020). A recent study has shown its potential protective role in reducing externalizing symptoms specifically among adolescents experiencing emotional distress (Royuela-Colomer et al., 2024).

Within this family system, the mother–child subsystem is particularly salient during early childhood. As primary caregivers with greater involvement and responsibility for children’s daily care and emotional needs, mothers may uniquely shape children’s externalizing problems through supportive and permissive parenting (e.g., warmth, responsiveness, and low demandingness) (Cox & Paley, 1997; Rinaldi & Howe, 2012). For example, maternal mindful parenting has been demonstrated to reduce adolescents’ externalizing behaviors (Larrucea-Iruretagoyena et al., 2024; W. Yang et al., 2022). In particular, it is reasoned that maternal mindful parenting may play an even more pronounced role in relation to externalizing behaviors during early childhood (Han et al., 2021; Siu et al., 2016).

Does maternal mindful parenting reduce externalizing problem behaviors in preschool children with CU traits? The Risk-Buffering Model (RBM) proposes that protective factors can weaken the negative effects of risk factors (Fergus & Zimmerman, 2005; Hollister-Wagner et al., 2001). By consistently providing warm and empathetic responses, mindful motherhood may serve as a buffer, mitigating the manifestation and exacerbation of CU traits. Prior research has shown that mindful motherhood is of particular significance for alleviating children’s psychopathological symptoms (Bogels et al., 2014; Meppelink et al., 2016). Further evidence has suggested that children with CU traits may exhibit differential sensitivity to certain maternal parenting styles, particularly in that maternal warmth and autonomy support can alleviate externalizing problems (Clark & Frick, 2018; Obando et al., 2024; Pasalich et al., 2014; Zhu et al., 2024).

Based on the above research, this study proposes that maternal mindful parenting moderates the positive relationship between CU traits and EPBs in preschool children (Hypothesis 3).

### 1.4. The Current Study

Prior research has established a link between household chaos and the development of externalizing problem behaviors in early childhood. CU traits serve as an early marker of externalizing symptoms, yet it remains unclear whether these traits reflect a basic adaptive response to adverse contextual stimulation within chaotic home environments. Furthermore, considering that mothers play a critical role in early childhood development, whether maternal mindful parenting can mitigate externalizing problem behaviors in children with CU traits exposed to chaotic home environments remains to be further examined. Therefore, grounded in the Family Stress Model and developmental psychopathology, this study investigates the associations between household chaos and CU traits on preschool children’s externalizing problems, as well as the moderating role of maternal mindful parenting, by testing a moderated mediation model. This research contributes to a deeper exploration of the mechanisms underlying early behavioral problems and offers practical guidance for parenting.

## 2. Materials and Methods

### 2.1. Participants and Procedure

Electronic questionnaires were administered using a convenience sampling methodology through an online survey platform. A total of 615 mothers of children aged 3–7 years were recruited. Specifically, 84 children (13.7%) were aged 3–4 years, 211 (34.3%) were aged 4–5 years, 183 (29.8%) were aged 5–6 years, and 137 (22.3%) were aged 6–7 years. The children comprised 341 boys (55.4%) and 274 girls (44.6%). Participants were recruited with the assistance of kindergarten teachers, who distributed the questionnaire link to mothers of children aged 3–7 years. Eligible mothers were invited to complete the questionnaires and to report on their family, their child, and themselves. The inclusion criteria were as follows: (1) mothers of children aged 3–7 years; (2) clear consciousness and the ability to complete questionnaires independently; and (3) voluntary participation and signed informed consent. The exclusion criteria were as follows: (1) extremely short completion time; (2) regular response patterns; and (3) contradictory information. The study protocol was approved by the research ethics committee of Guangzhou University (Protocol Number: GZHU202301).

### 2.2. Measures

#### 2.2.1. Household Chaos

The Confusion, Hubbub, and Order Scale (CHAOS) comprises 15 items, each scored on a four-point Likert scale (never = 1, and always = 4) (Matheny et al., 1995). It evaluates the degree to which the daily home environment is marked by a lack of routine, disorder, and noise. Specifically, seven items pertain to routines and organization (e.g., “We have a regular routine at home first thing in the day”), while eight items reflect disorganization, confusion, and noise (e.g., “Our home is often in a state of fuss”). The Chinese version of this scale has been translated and standardized (Chang et al., 2016), and the recent study has shown the great reliability and validity in the Chinese context (Zhan & Guo, 2023; J. Zhao et al., 2023). In this study, the Cronbach’s α was 0.81.

#### 2.2.2. Callous–Unemotional Traits

The CU traits in young children were assessed using the Chinese version of The Inventory of Callous-Unemotional Traits (ICU) (Frick, 2004; Deng et al., 2016). The scale consists of 11 items (e.g., “He/she seems cold and inconsiderate”) and uses a 4-point Likert scale, ranging from “0 = completely inconsistent” to “3 = completely consistent”. The higher the total score, the lower the levels of empathy, compassion and guilt towards others in young children. Some studies have recently shown that this scale has great reliability and validity among Chinese preschool children (Cao et al., 2023; Tan et al., 2023; Zhu et al., 2024). In this study, the Cronbach’s α of the total ICU was 0.84.

#### 2.2.3. Externalizing Problem Behaviors

Based on previous research (Vuckovic et al., 2021), externalizing problems in young children were assessed using the hyperactivity and conduct problems subscales of the Strengths and Difficulties Questionnaire (SDQ). These two subscales capture core behavioral manifestations of externalizing problems in children, including impulsivity, hyperactivity, rule-breaking, and aggression, and the combination of these two subscales to index externalizing behavior is consistent with prior research (Roman et al., 2016; Vuckovic et al., 2021). The SDQ is a widely used and well-validated screening instrument with established validity in early childhood (Acar et al., 2019; Du et al., 2008; Roman et al., 2016). Ten items were rated using a 3-point Likert scale ranging from 0 = not true to 2 = certainly true. The higher the score, the more severe the degree of externalizing problems. In this study, the Cronbach’s α was 0.70.

#### 2.2.4. Maternal Mindful Parenting

The Interpersonal Mindfulness in Parenting Scale (IM-P) is utilized to assess the level of maternal mindfulness in parenting. The Chinese revision of the IM-P has been demonstrated to possess good reliability and validity among parents of Chinese preschool children (Li, 2022). The revised Chinese version of the IM-P consists of 16 items and encompasses four dimensions: compassion of the child, emotion regulation in parenting, acceptance of the child and full attention in parenting. The scale employs a 5-point Likert scale, ranging from 1 (never true) to 5 (always true). Higher scores reported by mothers indicate a greater degree of mindfulness in parenting. This scale has shown good reliability and validity in the previous study (Li, 2022). In this study, the Cronbach’s α of the IM-P reached 0.84.

### 2.3. Statistical Analysis

Data processing and all analyses were conducted using SPSS 27 and its macro programs. Initially, a Pearson correlation matrix was constructed to examine the bivariate correlations among variables, including household chaos, preschool children’s CU traits and externalizing behaviors, and maternal mindful parenting. Subsequently, two macro models were executed to construct a path model containing mediators and moderators. Model 4 was used to estimate the predictive effect of household chaos on the externalizing behaviors of young children, as well as the mediating effect of CU traits. Model 14 was employed to estimate the moderating role of maternal mindful parenting on the externalizing behaviors of young children with CU traits, particularly examining the positive alleviating effect of maternal mindful parenting. Thirdly, a total of 5000 bootstrap samples were generated, each with a sample size of 624, to calculate the standard errors and confidence intervals of the parameter estimates. If the 95% confidence interval does not include zero, the effect is considered statistically significant.

## 3. Results

### 3.1. Assessment of Common Method Bias

Because all study variables were assessed via maternal self-report, Harman’s single-factor test was used to examine common method bias (Podsakoff et al., 2003). All measurement items were subjected to factor analysis. The results showed that 12 factors had eigenvalues greater than 1, and the first factor accounted for 18.50% of the variance, which was below the 40% threshold. Therefore, no serious common method bias was present in the data of this study.

### 3.2. Descriptive Statistics and Correlation Analysis of Variables

Table 1 presents the means and correlations of the study variables. The results indicated that household chaos was significantly positively correlated with both CU traits (*r* = 0.33, *p* < 0.01) and EPBs (*r* = 0.40, *p* < 0.01) in young children. High levels of CU traits were significantly correlated with high EPBs (*r* = 0.43, *p* < 0.01). Maternal mindful parenting was significantly negatively correlated with both CU traits (*r* = −0.35, *p* < 0.01) and EPBs in children (*r* = −0.39, *p* < 0.01). Additionally, children’s age was significantly negatively correlated with CU traits (*r* = −0.10, *p* < 0.05). Children’s age was included as a covariate in subsequent analyses.

Notably, household chaos was strongly correlated with maternal mindful parenting (*r* = −0.61, *p* < 0.01); therefore, multicollinearity was examined using variance inflation factors (VIFs) and tolerance. All VIF values ranged from 1.01 to 1.69, and tolerance values ranged from 0.54 to 0.99. These estimates fell well within conventional thresholds (VIF < 5; tolerance > 0.20), indicating that multicollinearity exerted no substantive influence on the results.

### 3.3. CU Traits Acted as the Mediator

To test the mediating role of CU traits in the association between household chaos and EPBs, multiple regression analyses were conducted after controlling for age and gender as covariates. The results demonstrated that household chaos was positively associated with EPBs in young children (*β* = 0.40, *p* < 0.001). Next, the PROCESS Model 4 was used to test a mediation model with CU traits serving as a mediator. As shown in Table 2, when CU traits were considered as a mediator, the direct predictive effect of household chaos on externalizing problem behaviors remained significant (*β* = 0.29, *SE* = 0.04, 95% Boot CI = [0.22, 0.36]). The indirect effect of household chaos on externalizing problems via CU traits was also significant (*β* = 0.11, *SE* = 0.02, 95% Boot CI = [0.08, 0.14]). Specifically, the mediating effect accounted for 27.05% of the total effect, indicating that CU traits played a partial mediating role in the link between household chaos and young children’s externalizing symptoms.

### 3.4. Maternal Mindful Parenting as the Moderator

To further examine whether maternal mindful parenting moderates the association between CU traits and EPBs, a moderated mediation model was constructed and tested using the PROCESS Model 14. As shown in Table 3, after controlling for children’s gender and age, household chaos was significantly positively associated with CU traits (*β* = 0.32, *p* < 0.001). Children’s gender was also significantly associated with CU traits (*β* = 0.15, *p* < 0.05), indicating that boys had higher levels of CU traits than girls. Children’s age was negatively associated with CU traits (*β* = −0.08, *p* < 0.05), suggesting that younger children exhibited higher levels of CU traits. Furthermore, household chaos was significantly positively associated with EPBs (*β* = 0.20, *p* < 0.001). This direct path coefficient was smaller than that observed in the simple mediation model (*β* = 0.29), likely because the moderated mediation model additionally included maternal mindful parenting and its interaction with CU traits, which are correlated with household chaos and thus share part of its explained variance in EPBs. CU traits also were positively associated with EPBs (*β* = 0.32, *p* < 0.001). Moreover, both maternal mindful parenting (*β* = −0.16, *p* < 0.001) and its interaction term with CU traits (*β* = −0.07, *p* < 0.05) were significantly and negatively linked to children’s externalizing problems. Thus, maternal mindful parenting moderated the association between CU traits and EPBs.

To compare the conditional indirect effect of CU traits at different levels of maternal mindful parenting, maternal mindful parenting was categorized into three levels: mean (M), mean plus one standard deviation (M + SD), and mean minus one standard deviation (M − SD). As presented in Table 4, the mediating effect of household chaos on young children’s externalizing problem behaviors was significant across all three levels of maternal mindful parenting: under the low (M − SD, *β* = 0.13, *BootSE* = 0.02, 95% Boot CI = [0.08, 0.18]), mean (M, *β* = 0.10, *BootSE* = 0.02, 95% Boot CI = [0.07, 0.14]), and high (M + SD, *β* = 0.08, *BootSE* = 0.02, 95% Boot CI = [0.05, 0.11]) conditions. Notably, as maternal mindful parenting levels increased, this conditional indirect effect gradually weakened. This indicates that the mediating effect of CU traits is significantly moderated by maternal mindful parenting.

**Figure 1:**
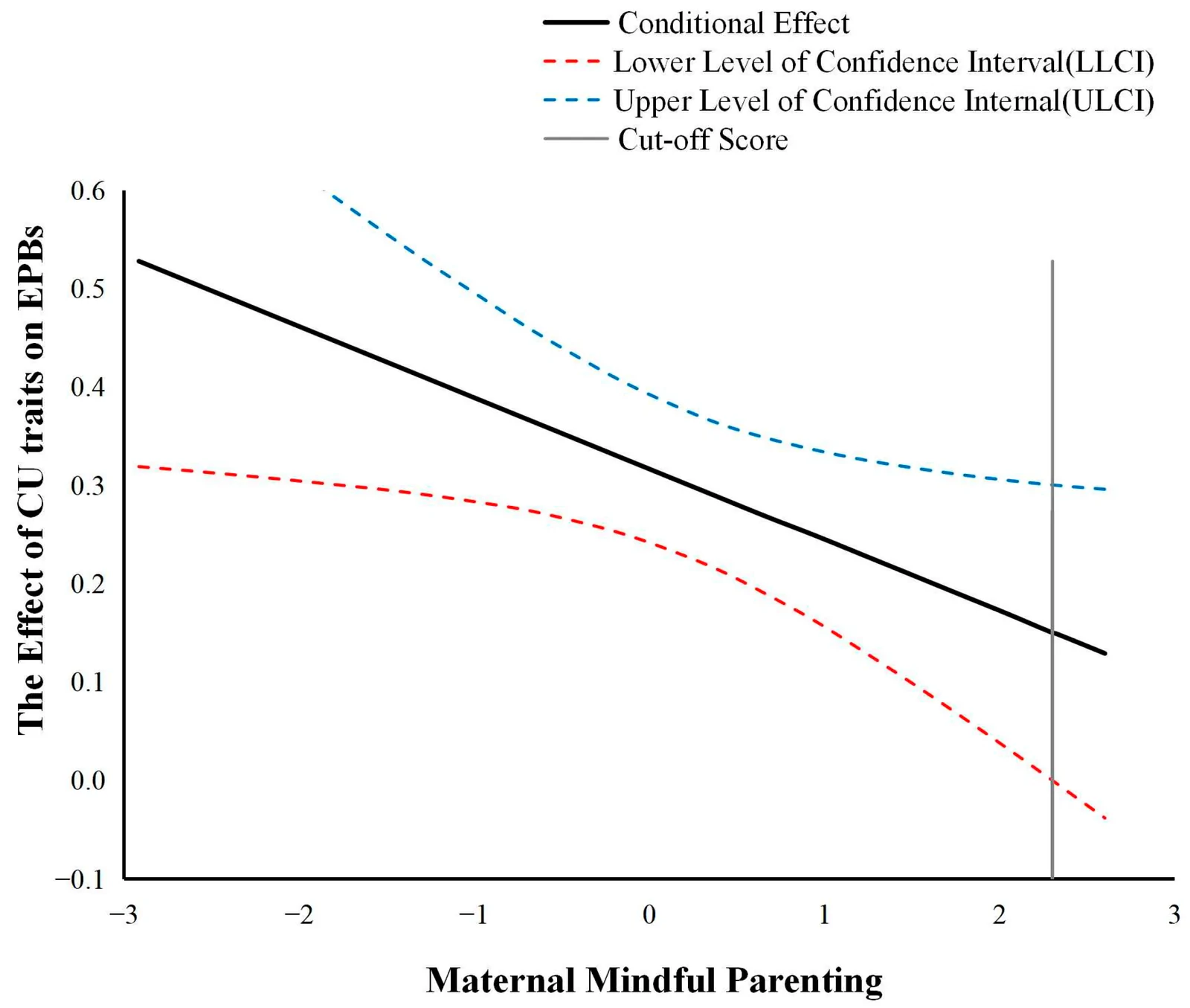
Johnson–Neyman plot of the moderating effect of maternal mindful parenting between CU traits and EPBs.

To further assess the moderating effect of maternal mindful parenting on the relationship between CU traits and externalizing problem behaviors, the Johnson–Neyman (J-N) technique was employed to determine the region of significance for the simple slope of CU traits on externalizing problem behaviors (Hayes, 2013). As shown in Figure 1, the simple slope was significant when the standardized score of maternal mindful parenting was within the range of −2.915 to 2.301. Specifically, when the standardized score of maternal mindful parenting was below 2.301, the positive association between CU traits and EPBs in young children weakened as mindful parenting increased. This suggests that maternal mindful parenting serves as a protective factor in the pathway between CU traits and externalizing problems, supporting the hypothesized moderated mediation model.

## 4. Discussion

This study investigated the link between household chaos and externalizing problem behaviors in young children, along with the potential mediating and moderating mechanisms underlying this link. Results indicated that household chaos was not only positively associated with EPBs but also indirectly associated with them through children’s CU traits. Furthermore, maternal mindful parenting moderated the positive association between CU traits and externalizing behaviors.

### 4.1. Impact of Household Chaos on Externalizing Problems During Early Childhood

The present study found that household chaos had a significant positive influence on externalizing behaviors in preschool children, supporting Hypothesis 1 and aligning with the robust associations in prior research (Dong et al., 2023; Farbiash et al., 2014; Larsen & Jordan, 2020; Wilhoit et al., 2021). These findings suggest that a chaotic home environment may serve as a critical ecological context for child development, providing support for Bronfenbrenner’s ecological systems theory (Bronfenbrenner, 1986). More broadly, household chaos can be conceptualized as a form of chronic family stress. According to the Family Stress Model (Conger & Donnellan, 2007), chronic family stress disrupts parenting, thereby impairing children’s adjustment. Household chaos can be viewed as a chronic, enduring form of family stress rather than a discrete acute stressor, making it a developmentally salient context for early externalizing problems.

In addition, a chaotic household is characterized by noise, crowding, and disorganization. Due to the lack of mature filtering strategies, young children’s cognitive resources are perpetually depleted in such an overstimulating environment, which could impair executive functioning and undermine self-regulatory capacity (Andrews et al., 2021; Liu et al., 2024; Vernon-Feagans et al., 2016). Such deficits are strong predictors of subsequent externalizing behaviors in children (Crespo et al., 2019; Y. Yang et al., 2022). Furthermore, unpredictable and disorderly environments make it difficult for young children to establish connections between behaviors and outcomes, thereby inducing frustration and helplessness and undermining their capacity to internalize social norms and develop appropriate behaviors (Evans & Stecker, 2004; Wachs, 1986). By contrast, family routines, by creating an environment characterized by structure, predictability, and clear boundaries, can effectively promote the development of self-regulation skills in young children and guide their behaviors toward appropriate and socially normative patterns (Bater & Jordan, 2017).

### 4.2. The Mediating Role of CU Traits

The findings of this study demonstrated that CU traits partially mediated the association between household chaos and EPBs in early childhood, confirming Hypothesis 2. Consistent with GAM and prior empirical evidence, CU traits can function as a mediator linking environmental adversity to individual problem behaviors (Anderson & Bushman, 2002; Carlson et al., 2015; Cutrín Mosteiro et al., 2016; Zhang & Zhu, 2023).

Consistent with the ACM (Del Giudice et al., 2012), household chaos was positively associated with CU traits in the present study. The theory holds that CU traits function as context-adaptive emotional strategies. Higher CU traits in chaotic families may signal adaptive calibration to early chronic stress. Emotional blunting and affective detachment enable children to withdraw from negative family interactions and reduce distress through self-protection (Fisher & Brown, 2018; Mills-Koonce et al., 2016). While adaptive within stressful family environments, this emotional profile is linked to socio-cognitive deficits and behavioral difficulties (Georgiou et al., 2019). Children with high CU traits, due to specific socio-cognitive deficits, show blunted perception of harm to others, reduced responsiveness to punitive cues, and poor behavioral inhibition, thereby increasing the risk of externalizing problem behaviors (Blair et al., 2001; Georgiou et al., 2019; Waller & Wagner, 2019).

Furthermore, children with CU traits and EPBs tend to become caught in mutually reinforcing negative exchanges with chaotic family environments. According to the Family Stress Model, chronic household chaos may deplete parents’ psychological resources and undermine parenting, making parents less responsive, less stimulating, and increasing their tendency to use harsher disciplinary practices (Cassinat et al., 2021; Conger & Donnellan, 2007; Masarik & Conger, 2017). This pathway, in turn, is linked to children’s CU traits and externalizing behaviors (Masarik & Conger, 2017; Mills-Koonce et al., 2016; Waller et al., 2012). At the child-driven level, children with early CU tendencies may be more challenging to parent and may evoke chaotic, inconsistent, less structured caregiving from overwhelmed parents (Brown et al., 2017; Flom et al., 2020; Hawes et al., 2011). For example, low responsiveness to warmth or discipline may disrupt routines and limits, increasing household chaos (Docherty et al., 2024; Hawes et al., 2011). Over time, children with CU traits tend to receive more chaotic and less structured parental care. Their CU traits and EPBs become maintained and reinforced, creating an escalating cycle (Oliver & Midouhas, 2023; Zhu et al., 2024).

### 4.3. The Moderating Role of Maternal Mindful Parenting

In this study, maternal mindful parenting significantly moderated the link between CU traits and externalizing behaviors in early childhood, supporting Hypothesis 3. Specifically, the more mothers engaged in mindful parenting, the fewer externalizing problems children with CU traits exhibited. This finding aligns with research on chronic environmental stress, which emphasizes that children’s adaptation trajectories are determined less by the objective severity of external stressors than by parental responses to adversity (Paryente & Gatenio Kalush, 2020; Pat-Horenczyk et al., 2017). Such a perspective helps explain the present moderation effect. Even under sustained chronic family-level stress such as household chaos, parental psychological resources may serve as critical protective factors that reshape risk pathways for vulnerable children. Against this background, the Risk-Buffering Model (RBM) provides further theoretical ground for understanding how maternal mindful parenting functions as a protective factor (Fergus & Zimmerman, 2005; Hollister-Wagner et al., 2001).

In addition, according to the mindful parenting model and related empirical research, mothers who practice mindful parenting are able to maintain present-moment sensitivity and acceptance toward their children’s emotions and behaviors even amid household chaos, thereby reducing automatic negative reactions and fostering more adaptive parenting strategies (Ahemaitijiang et al., 2021; Dumas, 2005; Lippold et al., 2015). These supportive parenting skills, in turn, help mitigate the children’s psychopathological symptoms (Bogels et al., 2014; Emerson et al., 2021; Meppelink et al., 2016). Furthermore, children with CU traits show differential sensitivity to maternal parenting practices (Clark & Frick, 2018; Kochanska et al., 2013; Obando et al., 2024; Pasalich et al., 2011). For example, a study of preschool children found that children with CU traits were less responsive to negative parenting (e.g., punitive discipline, inconsistency) but more responsive to positive parenting (e.g., warmth, positive reinforcement) (Clark & Frick, 2018). In the present study, higher maternal mindfulness may amplify the benefits of positive parenting and buffer risks such as household chaos. Mindful parenting can reduce parents’ automatic harsh and coercive reactions under family stress and foster a responsive and warm parent–child relational environment, from which children with CU traits may particularly benefit, thereby lowering the risk of subsequent externalizing problem behaviors. Moreover, although not central to our hypotheses, boys and younger children exhibited higher CU traits in this sample, consistent with prior evidence that CU traits are more prevalent among boys (Zhu et al., 2026; Zumbach et al., 2021) and may vary developmentally in early childhood (Ezpeleta et al., 2017). Future research should more systematically examine gender and age differences in CU traits during early childhood and test whether these differences remain consistent across different family environments.

### 4.4. Limitations and Future Research

The current study has several limitations. First, this cross-sectional study captured only concurrent associations, precluding conclusions about temporal precedence and leaving the possibility of reverse causality open. Given the malleability of CU traits in early childhood, future longitudinal research is needed to disentangle these bidirectional processes and examine their developmental trajectories across different environments. Second, externalizing problems were assessed with a brief SDQ composite, which may not capture the full range of externalizing behaviors and showed only marginal reliability. Future research should use more comprehensive and dimensional measures. Third, all variables in this study were assessed via maternal report, which may inflate the associations among variables. Future research should collect data from multiple informants (e.g., fathers, teachers, and children themselves where age-appropriate) and use multiple methods (e.g., observation and behavioral tasks) to further validate the findings. Fourth, this study lacked a formal SES measure. Because household chaos and parenting are strongly patterned by socioeconomic conditions, observed associations may partly reflect unmeasured SES differences. Future research should include validated SES indicators (e.g., income-to-needs ratio, maternal education, occupational prestige) as covariates or moderators. Fifth, all participants were recruited from Guangdong Province, China, and the findings may not generalize to populations with different cultural norms around parenting, child behavior, or household chaos. Future studies should test these associations across diverse cultural contexts.

## 5. Conclusions

Drawing on an integrated framework combining the Family Stress Model and developmental psychopathology, a moderated mediation model was employed in the current study to investigate the developmental mechanisms of EPBs among Chinese preschoolers. Results suggested that household chaos was not only directly associated with EPBs but also indirectly associated with them through CU traits. Notably, maternal mindful parenting moderated the association between CU traits and EPBs. These findings highlight the need for targeted prevention and intervention focusing on home-environment improvement and maternal mindful parenting training, particularly among families with young children at socioemotional and behavioral risk. Future research should include fathers and other caregivers as informants to better capture the association between household chaos and early childhood development. Longitudinal designs would help clarify the direction of these associations and trace the developmental trajectories of CU traits and EPBs across different contexts over time.

## Figures and Tables

**Table 1:** Descriptive statistics and correlations among variables.

Variables	*M*	*SD*	1	2	3	4	5	6
1 Gender	0.55	0.50	—					
2 Age	4.61	0.98	0.03	—				
3 Household chaos	2.03	0.36	−0.02	−0.04	—			
4 CU traits	1.96	0.35	0.07	−0.10 *	0.33 **	—		
5 EPBs	1.61	0.29	0.04	−0.06	0.40 **	0.43 **	—	
6 Maternal mindful parenting	3.73	0.49	−0.01	−0.05	−0.61 **	−0.35 **	−0.39 **	—

*N* = 615. Gender was a virtual encoding variable, Boy = 1, Girl = 0. CU traits, callous–unemotional traits; EPBs, externalizing problem behaviors. * *p* < 0.05. ** *p* < 0.01.

**Table 2:** Testing of the mediating role of CU traits.

Path	*β*	*SE*	*t*	95% CI	
				LLCI	ULCI
Household chaos → EPBs	0.29	0.04	7.83 ***	0.22	0.36
Household chaos → CU traits	0.32	0.04	8.52 ***	0.25	0.40
CU traits → EPBs	0.33	0.04	8.91 ***	0.26	0.40
The indirect effect	0.11	0.02		0.08	0.14

*N* = 615. All variables in the model were standardized before being entered into the regression equation. CU traits, callous–unemotional traits. EPBs, externalizing problem behaviors. *** *p* < 0.001.

**Table 3:** Testing of the moderating role of maternal mindful parenting.

Predictor	Model 1: CU Traits				Model 2: EPBs			
	*β*	*SE*	*t*	95%CI	*β*	*SE*	*t*	95%CI
Household chaos	0.32	0.04	8.52 ***	[0.25, 0.40]	0.20	0.04	4.55 ***	[0.11, 0.29]
CU traits					0.32	0.04	8.29 ***	[0.24, 0.39]
Maternal mindful parenting					−0.16	0.03	−3.49 **	[−0.24, −0.07]
Maternal mindful parenting × CU traits					−0.07	0.03	−2.27 *	[−0.14, −0.01]
Gender	0.15	0.08	2.04 *	[0.00, 0.30]	0.05	0.07	0.68	[−0.09, 0.18]
Age	−0.08	0.04	−2.15 *	[−0.16, −0.01]	−0.04	0.04	−0.97	[−0.10, 0.04]
*R* ^2^	0.12				0.28			
*F*	27.22 ***				39.63 ***			

*N* = 615. Gender was a virtual encoding variable, Boy = 1, Girl = 0. CU traits, callous–unemotional traits. EPBs, externalizing problem behaviors. * *p* < 0.05, ** *p* < 0.01, *** *p* < 0.001.

**Table 4:** Mediating effect size at different levels of maternal mindful parenting.

Different Levels	Effect	BootSE	BootLLCI	BootULCI
M − SD	0.13	0.02	0.08	0.18
M	0.10	0.02	0.07	0.14
M + SD	0.08	0.02	0.05	0.11

## Data Availability

The original contributions presented in this study are included in the article. Further inquiries can be directed to the corresponding author.
